# Long-Term Results of Complex Abdominal Aortic Aneurysm Open Repair

**DOI:** 10.3390/jpm12101630

**Published:** 2022-10-01

**Authors:** Yamume Tshomba, Simona Sica, Fabrizio Minelli, Marco Ferraresi, Chiara de Waure, Tommaso Donati, Francesca De Nigris, Claudio Vincenzoni, Francesco Snider, Giovanni Tinelli

**Affiliations:** 1 Unit of Vascular Surgery, Fondazione Policlinico Universitario A. Gemelli IRCCS—Università Cattolica del Sacro Cuore, 00168 Rome, Italy; 2 School of Vascular Surgery, University of Milan, 20122 Milan, Italy; 3 Dipartimento di Medicina e Chirurgia, Università degli Studi di Perugia, 06123 Perugia, Italy

**Keywords:** complex abdominal aortic aneurysm, pararenal, paravisceral, open surgery, renal decline, personalized medicine

## Abstract

This study investigated the long-term outcomes of patients treated with open surgical repair for complex abdominal aortic aneurysms (c-AAAs). A total of 119 patients with c-AAAs undergoing repair between January 2010 and June 2016 in a high-volume aortic center were included. The long-term imaging follow-up consisted of yearly abdominal ultrasound examinations and 5-year computed tomography angiography. At a median follow-up of 76 months (IQR 38 months), forty-three deaths (37%) and three (2.5%) aortic-related deaths were observed. Long-term chronic renal decline was observed in fifty (43.8%) patients, significantly correlated with post-operative acute kidney injury. During the follow-up, five reinterventions (4.3%) were performed. The present study suggests that open c-AAA repair can be performed with acceptable operative risk with durable results. To achieve the best possible long-term outcome, the open surgery repair of complex AAA should be performed in high-volume aortic centers and tailored to the patient.

## 1. Introduction

The advent of endovascular technologies has revolutionized the treatment of the complex abdominal aortic aneurysm (c-AAA) [1]. However, open surgery repair (OSR) remains the standard of care for patients with c-AAA “fit for surgery”. The open repair of c-AAAs is challenging given the extended visceral mobilization, possible mesenteric ischemia, substantial effect on cardiocirculatory function, and renal ischemia, with possible renal dysfunction [2,3,4].

The aim of this study is to analyze the long-term outcomes of complex abdominal aortic aneurysm open repair.

## 2. Materials and Methods

### 2.1. Study Population

This retrospective cohort study analyzes the outcomes of all consecutive patients treated for c-AAA between January 2010 and June 2016 from the Unit of Vascular Surgery of Fondazione Policlinico Universitario A. Gemelli IRCCS (FPUAG; Rome, Italy). Consecutive patients who underwent open surgery repair by a single experienced operator (>70 elective AAA repairs per year) in the high-volume center were prospectively collected during the study period. Complex AAAs are defined as aneurysms that involve the renal or mesenteric arteries and extend up to the level of the celiac axis or diaphragmatic hiatus but do not extend into the thoracic aorta, including juxtarenal aortic aneurysms (jr-AAA) (extend to but do not involve the renal arteries and necessitate suprarenal aortic clamping), pararenal aortic aneurysms (involve at least one of the renal arteries and extend up to but not cephalad to the superior mesenteric artery (SMA)), paravisceral aortic aneurysms (involve the renal arteries and SMA but not the celiac axis), and extent IV thoraco-abdominal aortic aneurysm (proximal extension of the aneurysm to the celiac axis or diaphragmatic hiatus) [1]. The study population is the unmatched OSR arm of a previous paper published in 2018 [5].

The study was performed in accordance with the Institutional Ethics Committee rules and approved by the ethics review boards of FPUAG (ID: 1843). Individual consent was waived. The data were retrospectively and anonymously analyzed. All patients provided consent for the intervention.

Patients were observed with regular postoperative appointments. Abdominal ultrasound examination was performed at 3 months and yearly thereafter. Computed tomography (CT) angiography was performed if abnormalities were found on ultrasound examination. However, all the patients received an angio-CT 5 years after surgery in the case of normal creatinine values or a CT without contrast associated with a contrast-enhanced ultrasound in the case of abnormal creatinine levels. A survival assessment was completed by phone interview.

The study excluded patients treated for extent I to III thoracoabdominal aneurysms, ruptured or symptomatic aneurysms, and dissections or connective tissue disorder aneurysms.

### 2.2. Endpoints and Definitions

The primary endpoints of this study were the mortality rate, aortic-related mortality rate, and chronic renal decline (CRD) during follow-up. Secondary endpoints included the aortic-related reintervention rate, the target vessel occlusion rate, the proximal aorta degeneration, access-related complications, graft infection, and the presence of “de novo” thoraco-abdominal aortic disease during the follow-up. Hospitalizations for bowel obstruction, without a subsequent operation, were also recorded.

Aortic-related mortality was defined as any death that results from aneurysm rupture, aorta-related complications, or a complication of a secondary intervention.

The estimated glomerular filtration rate (eGFR) was determined by the abbreviated Modification of Diet in Renal Disease study equation (eGFR [mL × min^−1^ × 1.73 m^−2^] = 186 × [serum creatinine] ^−1.154^ × [age] ^−0.203^ × [0.704 if female] × [1.210 if African American]).

Preoperative renal function was estimated using the chronic kidney disease (CKD) staging system and stratified as normal (stages 1 and 2, eGFR **≥** 60 mL/min/1.73 m^2^) and abnormal (stage 3–4, eGFR < 60 mL/min/1.73 m^2^). Patients in stage 5 (eGFR < 15 mL/min/1.73 m^2^ or dependent on renal replacement therapy) were excluded from the renal function analysis.

A postoperative deterioration of the eGFR by 25% within 1 week according to the RIFLE classification (Risk, Injury, Failure, Loss of kidney function, and End-stage renal failure) was defined as AKI. Severe AKI was defined as a >50% decrease in eGFR [6].

Chronic renal decline was defined in patients with normal (stage 1–2) preoperative renal function as a reduction in the eGFR to <60 mL/min/1.73 m^2^ during follow-up. In patients with abnormal function (stages 3 and 4) preoperatively, it was defined as an eGFR reduction of >20% or de novo dependence on permanent renal replacement therapy [7].

Aortic-related reintervention included all secondary interventions related to the initial procedure or to the graft and its target vessels during follow-up, as defined by the reporting standards [1].

Target vessel occlusion was defined as complete obstruction of the artery with no evidence of flow. Proximal aorta degeneration was defined as a diameter increase of > 5 mm within 5 cm above the proximal anastomosis. Access-related complications included any incisional hernia or bulging abdominal wall.

### 2.3. Surgical Procedure

A retroperitoneal approach was usually performed with a left flank incision according to Williams et al. [8] and Sicard et al. [9]. The retroperitoneal exposure of the aorta was achieved through an oblique incision from the tip of the eleventh rib to the lateral rectus border at the paraumbilical level. The anatomic access to the aorta was completed with the position of the left kidney (anterior or posterior) during the procedure, depending on the level of aortic clamping and the left renal vein anatomy. Exposure of the proximal abdominal aorta was obtained by division of the left diaphragmatic crus. Different reconstruction strategies were performed: Proximal beveled anastomosis including renal/visceral vessels; proximal end-to-end anastomosis and direct renal reattachment to the aortic graft; and proximal end-to-end anastomosis and uni- or bilateral aorto-renal or aorto-visceral bypass with an 8-mm Dacron graft interposition. All renal and visceral anastomoses were performed with a 6/0 polypropylene running suture. Proximal and distal aortic anastomosis was performed with a 3/0 polypropylene running suture. In the case where revascularization of the renal artery was performed, a selective perfusion of the renal arteries was achieved through the infusion of cold (4 °C) lactated Ringer solution and mannitol 10%.

### 2.4. Statistical Analysis

Means and medians with their 95% confidence intervals (95%CI) were used to describe quantitative variables and absolute and relative frequencies to report qualitative ones. Follow-up length was reported as the median with the interquartile range (IQR). Mortality, reintervention, and renal function decline were analyzed by means of Kaplan–Meier curves. Patients that died during the hospital stay were excluded from the long-term analysis of reintervention and renal function decline. Similarly, patients with hemodialysis were excluded from the analysis of renal function decline. The log-rank test was used to assess differences in time to renal function with respect to the presence of AKI and pre-operative renal function (1–2 vs. 3–4). A subgroup analysis was performed in patients with AKI to investigate differences in time to renal decline regarding the severity of AKI and the recovery of renal function at hospital discharge. Multivariable logistic regression was performed to assess the role of age, sex, clamp level, clamp duration (≤30 min vs. >30 min), visceral repair, and pre-operative renal function in predicting post-operative AKI. All the variables were entered into the model, and the backward stepwise process was used to identify the best model to explain the data. Results were reported as the Odds Ratio (OR) with 95%CI. The *p*-value was set at 0.05, and Stata 14 and SPSS 22 were used to perform the analysis.

## 3. Results

### 3.1. Demographic Data

Between January 2010 and June 2016, 119 elective conventional open repairs for c-AAA were carried out at our center. Demographics and baseline characteristics are shown in Table 1.

### 3.2. Procedure Data

**Figure 1:**
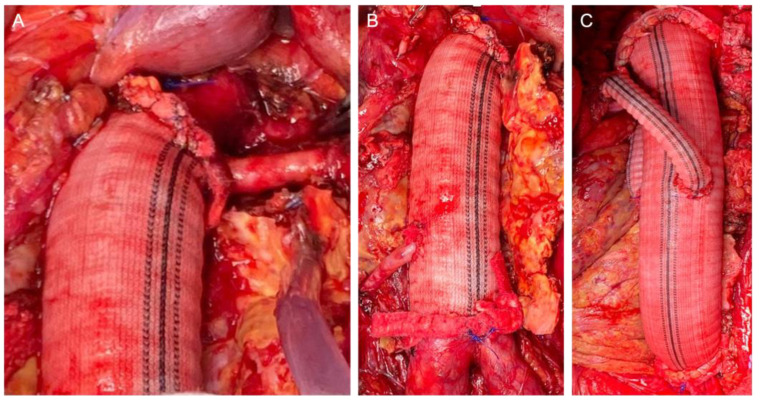
Intraoperative details of the reconstruction strategies: Proximal beveled anastomosis including the renal artery (**A**); direct renal reattachment to the aortic graft (**B**); bilateral aorto-renal bypass (**C**).

The retroperitoneal approach was performed in 105 (88.2%) patients, whereas the transperitoneal approach was performed in 14 (11.8%). The clamp level was inter-renal in 12 (10.1%) cases, suprarenal in 58 (48.7%) cases, and supra-visceral in 49 (41.2%) cases, of which 24 (20.2%) were above the SMA and 25 (21.0%) were supra-celiac. The mean operating time was 312.1 ± 77.9 min. The mean proximal aortic clamping time was 27.5 ± 8.3 min. The mean proximal aortic clamping time was 26.8 ± 7.3 and 28.6 ± 9.3 min in the case of supra-renal and supravisceral clamping, respectively. We performed 27 associated renal and visceral procedures in 21 patients (20.5%): 15 renal artery reimplantation (including polar renal arteries), 6 aortorenal bypasses, 2 aorto-visceral bypasses (SMA and common hepatic artery), 2 renal angioplasties (inclusive proximal anastomosis with a beveled graft), and 2 renal transaortic endarterectomies. In these cases, the mean proximal aortic clamping time was 33.8 ± 11.2 min. Intraoperative details of the aortic reconstruction strategies are presented in Figure 1.

### 3.3. Early Results

The median post-operative hospital stay was 13 days (IQR 39 days) with a median closed intensive care unit stay of 1 day (IQR 10 days).

The 30-day mortality rate was 1.7% (*n* = 2), and two patients died after OSR of myocardial infarction and multiorgan failure, respectively. In-hospital mortality was 2.5% (*n* = 3), and one additional patient died of myocardial infarction. Cardiac and pulmonary complications were observed in 9 (7.7%) and 14 (11.8%) patients, respectively. No spinal cord ischemia occurred. No cases of intestinal ischemia, bowel resection, and ischemic liver dysfunction were observed in the post-operative period.

There were seven (5.9%) reinterventions: Five wound dehiscence (surgical revision) and two retroperitoneal hematomas (surgical revision). No renal or bypass occlusions occurred during hospitalization.

AKI was observed in 64 (53.8%) patients, of whom 49 (76.6%) and 15 (23.4%) presented preoperative normal and abnormal renal function, respectively.

Twenty (31.2%) cases of AKI were severe. Four (3.4%) patients required dialysis immediately after the procedure, of which two (1.7%) cases were permanent dialysis. In the 64 patients with AKI after OSR, 41 (64.1%, 29 normal vs. 12 abnormal preoperative renal function) returned to preoperative eGFR levels before discharge. In the multivariable analysis, clamp duration of >30 min (OR 1.05; 95%CI 1.01,1.10; *p* = 0.03) and visceral repair (OR 2.21; 95%CI 0.98, 4.96; *p* = 0.06) were significant predictors of the time-dependent risk of postoperative AKI.

### 3.4. Long-Term Results

The median follow-up was 76 months (IQR 38 months). Forty-three deaths (37%) were observed. The cause of death was cardiovascular complications in 15 (12.9%) cases, followed by cancer in 11 (9.2%) patients. The survival rate was 83.2% ± 3.4%, 73.1% ± 4.1%, and 54.7% ± 6.2% at 3, 5, and 8 years, respectively.

There were three (2.5%) aortic-related deaths during follow-up. Two (1.7%) patients died from graft infection, and one (0.8%) from iliac pseudoaneurysm rupture. Aortic-related survival was 97.5% ± 1.4% at 5 years and 93.1% ± 3.4% at 8 years.

**Figure 2:**
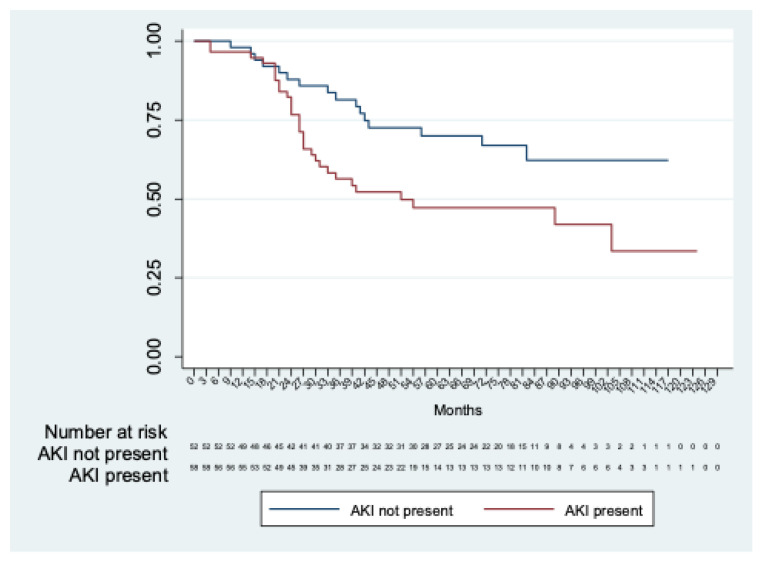
Kaplan–Meier analysis of freedom from chronic renal decline in patients with AKI vs. patients without AKI.

Long-term renal function was estimated in 114 patients, excluding three in-hospital deaths and two patients with permanent dialysis. Chronic renal decline was observed in fifty (43.8%) patients. The mean time to renal decline was 76.7 months (95% CI 67.2; 86.2). A significant difference (*p* = 0.01) in time to renal function decline was observed between patients with and without AKI (mean time to renal decline: 86.4 months (95%CI 74.5; 98.3) in patients without AKI and 65.9 months (95%CI 53,0; 78,8) in patients with AKI) (Figure 2).

**Figure 3:**
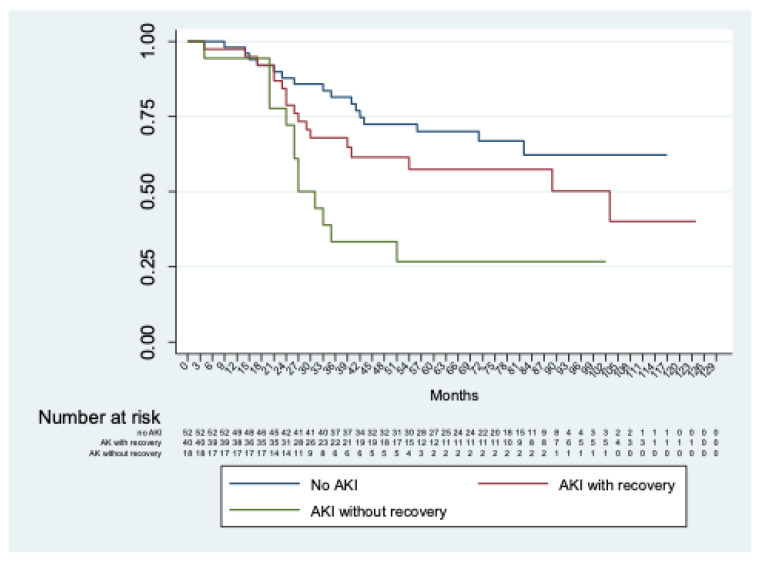
Kaplan–Meier analysis of freedom from chronic renal decline of patients without AKI vs. with AKI with and without recovery of renal function at discharge.

Considering only patients with AKI, no significant differences (*p* = 0.13) were observed between those with or without severe AKI. In addition, significant differences (*p* < 0.01) were shown comparing patients without AKI with those with AKI with and without recovery of renal function at discharge (Figure 3).

No significant differences (*p* = 0.71) were observed in the comparison between patients with pre-operative renal function stages 1–2 vs. 3–4. Renal function analysis is reported in Table 2.

Aortic-related reintervention was performed in five patients (4.2%), including three (2.5%) distal pseudoaneurysms of the common iliac arteries and one (0.8%) of the femoral artery, and one (0.8%) removal of an infected prosthetic graft. Reintervention-free survival was 96.9% ± 1.7% at 5 years and 93.7% ± 2.8% at 8 years.

There were five (4.2%) renal artery occlusions, including one native renal artery, two reimplanted renal arteries, and two aorto-renal bypasses. No visceral artery occlusion was observed.

Proximal aorta degeneration was observed in 15 cases (12.6%), of which one showed the indication for treatment.

Access-related complications were observed in 30 (25.2%) cases, including 10 (8.4%) incisional hernias and 20 (16.8%) bulging abdominal walls. None of these cases of incisional hernia and abdominal bulging wall had an indication for treatment. No cases of hospitalization for bowel obstruction were observed.

During follow-up, there were two (1.7%) cases of graft infection. De-novo aortic pathology was observed in eight (6.7%) patients: Iliac aneurysm in three (2.5%) cases, thoracic penetrating aortic ulcer in three (2.5%) cases, and thoracic aneurysm in two (1.7%) cases.

## 4. Discussion

In recent decades, fenestrated and branched endovascular aortic repair (F-BEVAR) has been increasingly utilized to treat complex aneurysm with challenging necks and the involvement of renal and visceral arteries and represent an effective and safe alternative to OSR. However, OSR remains the gold standard for patients “fit” for surgery.

In the current series, perioperative mortality was low (2.5%) and it was comparable to that reported in the literature. Tallarita et al. [10] reported mean mortality of 4.1% (0–11%). Jongkind et al. [11] reported a pooled mortality rate of 2.9%.

C-AAA OSR is characterized by a period of renal ischemia due to proximal aortic clamping, potentially increasing the risk of postoperative AKI. Jongkind et al. [11] analyzed 21 studies regarding juxtarenal abdominal aortic aneurysms and reported a median postoperative acute renal dysfunction of 18% with high variability between studies (0–39%) and a cumulative incidence of new onset dialysis of 3.3%. Tallarita et al. [10] analyzed 30 articles covering pararenal (pr) AAA OSR in 2228 patients and reported that the average rate of AKI was 31% (2–40%) and the rate of permanent dialysis was 3%. Kabbani et al. [12] observed a rate of AKI of 60% and a rate of new onset dialysis of 4%. The incidence of AKI in the present study was 53.8% and it was apparently higher than what has been reported previously. Nevertheless, the rate of new onset and permanent dialysis was similar to the other reports (3.4% and 1.7%, respectively), and only 16% of the patients had persistent renal impairment at discharge. Unfortunately, previous authors have used a variety of definitions to report AKI and, thus, an accurate comparison is difficult. Our high rate of AKI is likely due to the strict application of the RIFLE classification that provides a very sensitive definition of AKI. In addition, the high rate of supra-visceral clamping (41.2%) and renal revascularization procedures (22.7%) reported in the current series might also contribute to explaining this result.

In our analysis, clamp duration > 30 min (*p* = 0.03) and visceral repair (*p* = 0.06) were significant predictors of the time-dependent risk of postoperative AKI. Other authors reported a significant association between AKI and the clamp level, renal-visceral ischemia time, type of aneurysm, intraoperative renal perfusion, left renal vein division, pre-existing CKD, age, and diabetes [13,14,15,16].

Our results reflect the outcomes of a complex procedure performed by a single experienced operator in a high-volume aortic center. The early results are in line with the current literature. However, long-term outcomes remain poorly defined in the literature. Most of the reports focus on perioperative and mid-term outcomes of c-AAA OSR. In the present study, we reviewed our series of OSR of c-AAA analyzing long-term outcomes with a median follow-up of 76 months. Long-term survival rates at 5 and 8 years were 73.1% and 54.7%. These results were similar to long-term survival estimates in other series [12,17]. Three aortic-related deaths were observed, two of which were caused by graft infection.

However, long-term outcomes remain poorly defined, especially in terms of late renal function. Sugimoto et al. [3] reported a rate of chronic renal decline of 15.9% after pr-AAA and jr-AAA OSR, strictly correlated to the preoperative CKD stage. Notably, in their series, the rate of supra-visceral clamping was low (5.6%) and only 13% of the patients underwent renal artery-associated procedures. Chaufour et al. [16] analyzed the outcomes of 315 consecutive jr-AAA OSRs and reported a low rate of CRD (7.9%) during a mean follow-up of 4.3 years. They demonstrated that AKI was the strongest predictor of CRD, followed by diabetes and preoperative CKD. In the current series, CRD occurred in 43.9% of the patients during a mean follow-up of 76 months. In line with Chaufour et al. [16], AKI was a predictor of CRD. On the contrary, AKI was not correlated to preoperative CKD. The major credit of the present study was demonstrating that patients who developed AKI and recovered before discharge had a significantly lower risk of CRD compared to those who did not recover from AKI, but a significantly higher risk compared to those that did not develop AKI. We believe that AKI followed by complete recovery might still predispose individuals to late CRD, reducing renal function reserve. Another hypothesis is that this subgroup of patients has a reduced preoperative renal function reserve that predisposes them to both AKI and late CRD. This finding supports the idea that postoperative AKI should be considered a risk factor for late CRD even when the patient recovers completely from the acute damage. With the increasing application of F-BEVAR for c-AAA treatment, a comparison between open and endovascular repair in terms of CRD is needed.

The long-term advantage of open repair lies in very low reintervention rates. Our rate of reintervention during follow-up was 4.2%, including three distal pseudoaneurysms of the common iliac arteries and one of the femoral artery, and one removal of an infected prosthetic graft. In a recent metanalysis comparing OSR and F-EVAR for jr-AAA, Rao et al. [18] reported a pooled rate of reintervention after OSR of 4.9%. They also demonstrated that F-EVAR is associated with a greater number of repeat procedures during follow-up. There are now abundant data in the literature to support the idea that the trade-off between endovascular repair and OSR is durability and that F-B-EVAR patients remain at risk for reintervention indefinitely [19,20].

Our long-term complications included two graft infections (1.7%) and four anastomotic pseudoaneurysms (3.3%). Large cohorts with a significant follow-up pose the incidence of anastomotic pseudoaneurysms from 1% to 10% at 10 years [21,22]. Other complications described in the literature include occlusions (2%), aorto-enteric fistulas (1.6%), and prosthesis infections (1.3%) [23]. In this series, IHs were identified in 10 patients (8.4%). Chaufour et al. [16] reported a similar rate of IH (9.5%).

Throughout the analysis of the follow-up CT, we found that 15 patients (12.6%) presented aortic enlargement > 5 mm in the 5 cm above the proximal anastomosis. Nevertheless, only one patient presented a maximum diameter larger than 55 mm. One of the differences between OSR and F-BEVAR is that, in the latter, it is mandatory to cover a relatively long portion of the proximal aorta to achieve adequate sealing. Although proximal extension might compromise visceral vessels, it might also have a protective effect on aortic enlargement above the aneurysm sac. Nowadays, it is not clear whether extended aortic coverage protects from proximal aneurysmal degeneration or does any harm to visceral vessels. This topic must be further addressed.

The present study showed the results of one experienced surgeon in a high-volume aortic center, and this may be a limitation for reproducibility in a larger group of operators.

## 5. Conclusions

The present study describes the long-term outcomes of patients with c-AAA following OSR. Low rates of long-term aortic-related mortality and reintervention were observed. A high rate of CRD was observed and strictly correlated to post-operative AKI. Performing OSR of c-AAA in high-volume aortic centers is essential.

## Figures and Tables

**Table 1:** Baseline characteristics.

	*n* = 119 (%)
Age, mean (SD)	71.7 (6.8)
Male	109 (91.6)
Coronary artery disease	46 (38.7)
Chronic obstructive pulmonary disease	46 (38.7)
Diabetes Mellitus	12 (10.1)
Smoking	45 (37.8)
Preoperative renal function	
Stage 1–2	82 (68.9)
Stage 3–4	37 (31.1)
Aneurysm diameter, mm, mean (SD)	64.2 (13.5)
Anatomical classification	
Juxtarenal	37 (31.1)
Pararenal	57 (47.9)
Paravisceral	18 (31.6)
Type IV TAAA	7 (5.9)

**Table 2:** Renal function analysis.

	Acute Kidney Injury (AKI)	Severe AKI	Permanent Dialysis	Normal eGFR Levels before Discharge	Chronic Renal Decline during Follow-Up *
Cases (*n* = 119)	64 (53.8%)	18 (21.9%)	2 (1.7%)	41 (34.4%)	50 (43.8%) *

* Long-term renal function was estimated in 114 patients.

## Data Availability

All relevant data are within the paper.
